# The relationship between metabolic syndrome and its components with bladder cancer: a systematic review and meta-analysis of cohort studies

**DOI:** 10.4178/epih.e2022050

**Published:** 2022-05-30

**Authors:** Mozhgan Ahmadinezhad, Maedeh Arshadi, Elahe Hesari, Maedeh Sharafoddin, Hosein Azizi, Farzad Khodamoradi

**Affiliations:** 1 Department of Epidemiology and Biostatistics, School of Public Health, Tehran University of Medical Sciences, Tehran, Iran; 2 Research Center of Psychiatry and Behavioral Sciences, Tabriz University of Medical Sciences, Tabriz, Iran; 3 Department of Social Medicine, Faculty of Medicine, Ahvaz Jundishapur University of Medical Sciences, Ahvaz, Iran

**Keywords:** Urinary bladder neoplasm, Meta analysis, Metabolic syndrome, Metabolic syndrome components, Cohort studies

## Abstract

A previous meta-analysis, entitled “The association between metabolic syndrome and bladder cancer susceptibility and prognosis: an updated comprehensive evidence synthesis of 95 observational studies involving 97,795,299 subjects,” focused on all observational studies, whereas in the present meta-analysis, we focused on cohort studies to obtain more accurate and stronger evidence to evaluate the association between metabolic syndrome and its components with bladder cancer. PubMed, Embase, Scopus, and Web of Science were searched to identify studies on the association between metabolic syndrome and its components with bladder cancer from January 1, 2000 through May 23, 2021. The pooled relative risk (RR) and 95% confidence intervals (CI) were used to measure this relationship using a random-effects meta-analytic model. Quality appraisal was undertaken using the Newcastle-Ottawa Scale. In total, 56 studies were included. A statistically significant relationship was found between metabolic syndrome and bladder cancer 1.09 (95% CI, 1.02 to 1.17), and there was evidence of moderate heterogeneity among these studies. Our findings also indicated statistically significant relationships between diabetes (RR, 1.23; 95% CI, 1.16 to 1.31) and hypertension (RR, 1.07; 95% CI, 1.01 to 1.13) with bladder cancer, but obesity and overweight did not present a statistically significant relationship with bladder cancer. We found no evidence of publication bias. Our analysis demonstrated statistically significant relationships between metabolic syndrome and the risk of bladder cancer. Furthermore, diabetes and hypertension were associated with the risk of bladder cancer.

## INTRODUCTION

Metabolic syndrome is defined as a set of risk factors that jointly cause adverse outcomes, including type 2 diabetes mellitus and cardiovascular disease [1]. These risk factors are central obesity, insulin resistance, hypertriglyceridemia, hypercholesterolemia, hypertension, and reduced high-density lipoprotein (HDL) cholesterol concentrations [2]. According to epidemiological studies, the prevalence of metabolic syndrome varies from 20% to 45% of the population, and this rate is expected to increase to approximately 53% by 2035 [3-5]. Metabolic syndrome increases with age, and women are more susceptible than men [6]. Studies have consistently shown that metabolic syndrome is associated with an increased risk of several cancers [7-10]. Furthermore, each component of metabolic syndrome (including obesity, hypertension, hyperglycemia, and dyslipidemia) independently increases the risk of various cancers [11].

Bladder cancer has recently been linked to metabolic syndrome [12]. Bladder cancer is the 10th most prevalent cancer worldwide, and the incidence of bladder cancer is increasing. The International Agency for Research on Cancer reported 573,278 new cases and 21,253 bladder cancer deaths in 2018 [13]. It is also associated with significant complications and mortality in many parts of the world, and it therefore imposes a high burden of disease on communities [14]. Bladder cancer is a multifactorial disease, and evidence from clinical studies suggests that metabolic syndrome may increase the risk, recurrence, and mortality of this condition. Many studies have also shown that aging and obesity are risk factors for bladder cancer [15]. Therefore, older people with chronic diseases such as metabolic syndrome are more likely to be affected by bladder cancer [16].

Research has shown that low HDL levels are an important risk factor for urothelial carcinoma of the bladder. Furthermore, through excess secretion of insulin, metabolic syndrome could be associated with the malignant potential of urothelial carcinoma of the bladder [17]. In addition, an association between being overweight, which is a component of metabolic syndrome, and the risk of recurrence of bladder cancer has been reported [18]. Conflicting information has been reported regarding the association between metabolic syndrome and the risk of bladder cancer in various studies. Some cohort studies have reported no statistically significant association between the components of metabolic syndrome and bladder cancer risk [19-21], whereas other studies have found statistically significant associations [19,22,23]. Meta-analyses have found that metabolic syndrome significantly increased the risk of bladder cancer [9,24].

Considering the available data, the association of metabolic syndrome and its components, including obesity, hypertension, high blood triglycerides, low HDL cholesterol levels, and insulin resistance, with bladder cancer has yet to be well enough defined to reach reliable results. Furthermore, the previous meta-analysis published in 2018 [24] focused on all observational studies, including cross-sectional studies and case-control studies, which may have more bias and lower quality than cohort studies. In this study, we focused on cohort studies to obtain more accurate and stronger evidence for the association between metabolic syndrome and bladder cancer. We also added new studies and our search was broader than those of previous studies.

## MATERIALS AND METHODS

### Literature search strategy

Initially, to identify whether studies satisfied the inclusion criteria, we screened the titles and abstracts of all studies using EndNote 20, an update from version X9 (Clarivate Analytics, Philadelphia, PA, USA). A wide search was performed of several electronic databases including PubMed, Embase, Scopus, and Web of Science from January 1, 2000, through May 23, 2021. The search terms comprised the following keywords: “bladder cancer,” “bladder neoplasms,” “bladder carcinoma,” “bladder tumor-associated antigen,” “urothelial carcinoma,” “noninvasive bladder cancer,” “non-muscle-invasive bladder cancer,” “muscle-invasive bladder cancer,” “metabolic syndrome,” “metabolic syndrome components,” “metabolic abnormalities,” “metabolic syndrome x,” “insulin resistance,” “obesity,” “overweight,” “hypertension,” “dyslipidemia,” “diabetes mellitus,” “hypertriglyceridemia,” and “low HDL.” We also investigated the references of all articles to identify studies that were not included during the initial search. The following inclusion criteria were selected for meta-analysis: the study had a cohort study design, the primary outcome was the risk of bladder cancer (quantified as the relative risk [RR] and the 95% confidence interval [CI]) associated with metabolic syndrome, and the study was published in English. The exclusion criteria were articles of the following types: letters to the editor, case reports, intervention studies, case-control studies, cross-sectional studies, reviews, and meta-analyses.

### Study selection

Initially, to identify whether studies satisfied the inclusion criteria, we screened their titles and abstracts. For studies where this decision was difficult to make based only on their titles and abstracts, a full-text assessment was conducted. Two authors (MA and FK) screened the final full-texts, and after reading the fulltexts of all eligible articles, made a final decision for each study. In cases of disagreement, the third review author’s opinion was solicited, or the issue was resolved by discussion.

### Data extraction

A structured data extraction form was used. The extracted data included: the last name of the first author, publication year, country, study purpose, sample characteristics, sample size, exposure, mean age, gender, confounders, and definitions of the components of metabolic syndrome. Data were extracted independently by the same 2 review authors (MA and FK) who conducted the study selection.

### Evaluating the quality of articles

The quality of papers was assessed using the Newcastle-Ottawa Scale (NOS) designed for observational studies [25]. The NOS consists of 3 domains, including the selection of study groups, comparability of groups, and description of exposure and outcome. This quality assessment tool includes 8 items and star scores for each study in each domain. All items have 1 star except the comparability domain (the maximum score based on stars for comparability domain is 2). The total score of each of the articles was calculated. Then, all selected studies were categorized based on these levels: high (7-10), medium (5-6), or low quality (< 4). Two authors (MA and MS) reviewed the articles separately. The third author’s opinion was used to resolve any disagreements.

### Statistical analysis

Pooled RRs and 95% CIs were calculated to measure the association of metabolic syndrome and its components with the risk of bladder cancer using a random-effects meta-analytic model. We used adjusted estimates. We used the Cochran Q-test and the I^2^ statistic to evaluate heterogeneity. A subgroup analysis was carried out according to gender (men and women) and weight (obesity and overweight). To identify influential studies in the meta-analysis, leave-one-out sensitivity analysis was performed. Publication bias was determined by a funnel plot and the Begg and Egger tests. A p-value < 0.05 was considered to indicate statistical significance. All analyses were performed using Stata version 14 (StataCorp., College Station, TX, USA).

### Ethics statement

Ethical approval was not sought because this study was based on published articles and no human or animal intervention was performed.

## RESULTS

### Study characteristics

**Figure 1.**
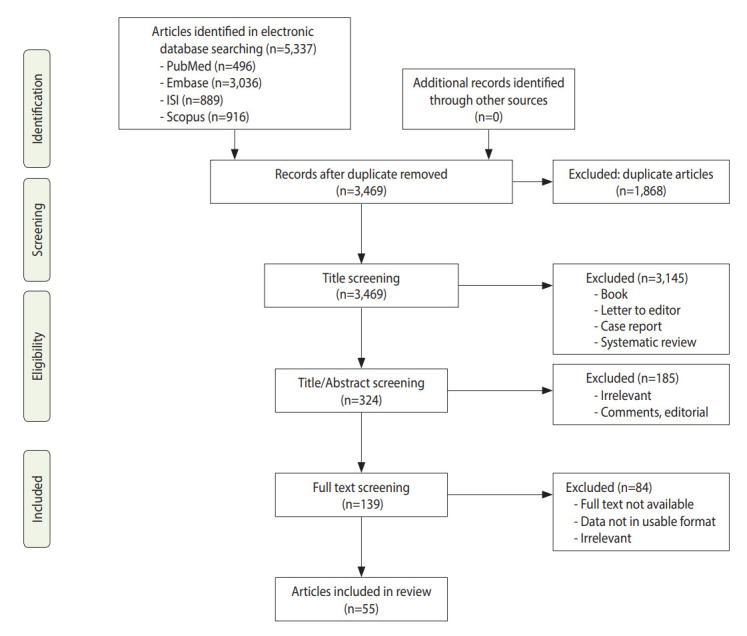
Flow chart depicting the study selection process (screening).

Figure 1 shows the search strategy and the algorithm of study selection. According to the keywords, MeSH (Medical Subject Headings) terms, and Emtree terms, 5,337 studies were identified. After considering the inclusion and exclusion criteria, relevant studies were retrieved and duplicates were removed. In this process, 3,145, 185, and 84 studies were excluded after screening their titles, abstracts, and full-texts, respectively. Finally, the quality of 55 related studies that satisfied the inclusion criteria was assessed. Of these, 11 studies were performed in the United States [23,26-35], 8 studies in Sweden [19,21,36-41], 8 studies in Korea [42-49], 6 studies in the United Kingdom [20,50-54], 7 studies in Taiwan [55-61], 2 studies each in China [62,63], Japan [64,65], Italy [66,67], Scotland [68,69], and Austria [70,71], and 1 study each in Iran [72], Norway [73], Netherland [74], European and Canada [75]. The cut-point score of 7 or higher was considered as indicating studies with high quality, and scores of 5-6 indicated moderate quality. Using these criteria, 33 studies had high levels of quality and 22 had moderate levels of quality (Supplementary Material 1). Supplementary Material 2 summarizes the characteristics of selected studies.

### Metabolic syndrome and bladder cancer risk

**Figure 2.**
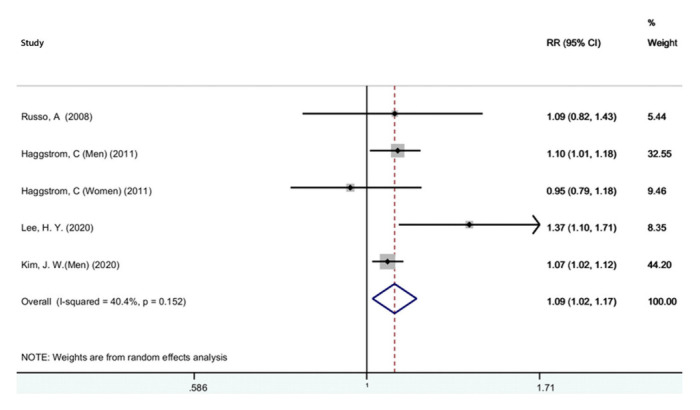
Forest plot of the association between metabolic syndrome and bladder cancer. RR, relative risk; CI, confidence interval.

Figure 2 shows the results of the random-effects meta-analysis and the adjusted pooled RR from the 4 studies included for the association of metabolic syndrome and bladder cancer [19,45,59,67]. The results showed a statistically significant relationship between metabolic syndrome and bladder cancer (RR, 1.09; 95% CI, 1.02 to 1.17). Furthermore, there was moderate heterogeneity between these studies (I^2^=40.4%; p=0.152). A sensitivity analysis showed that no single study significantly changed the pooled RR. Based on the Begg (p=0.851) and Egger (p=0.801) tests, we found no evidence of publication bias.

### Diabetes and bladder cancer risk

**Figure 3.**
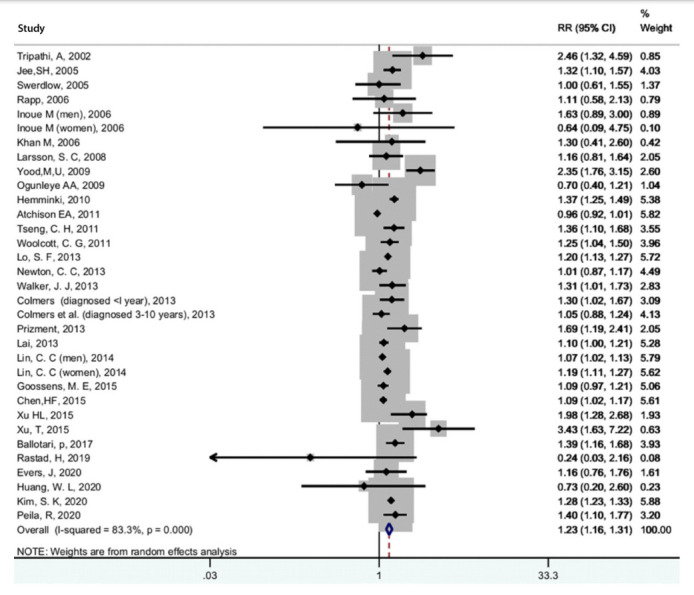
Forest plot of the association between diabetes and bladder cancer. RR, relative risk; CI, confidence interval.

**Figure 4.**
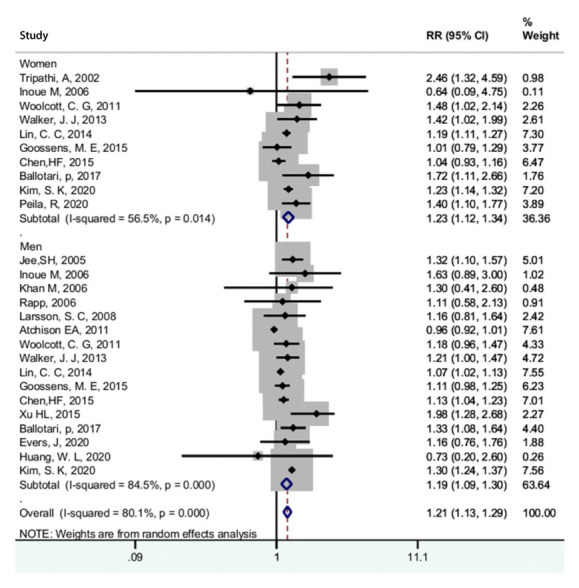
Forest plot of the association between diabetes and bladder cancer by gender. RR, relative risk; CI, confidence interval.

**Figure 5.**
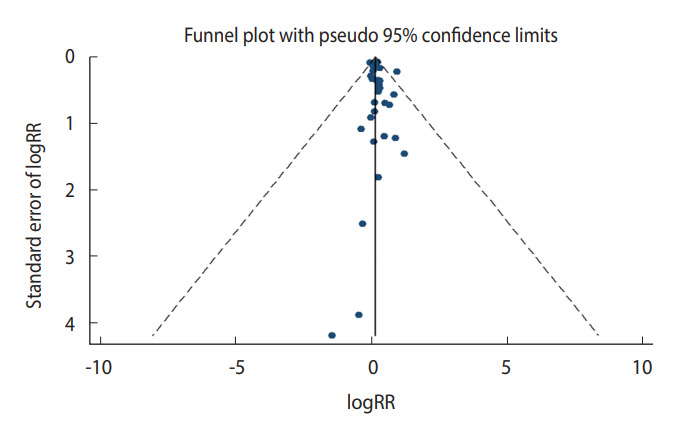
Funnel plot for publication bias regarding the association between diabetes and bladder cancer. RR, relative risk.

Figure 3 presents the adjusted pooled RR for the association of diabetes and bladder cancer. Thirty studies were included [20-22,28-31,34,41,46,52,53,55-58,61-66,68,69,71,72,74-76]. Based on this figure, diabetes increased the risk of bladder cancer (RR,1.23; 95% CI, 1.16 to 1.31). However, there was significant heterogeneity between studies (I^2^=83.3%; p<0.001). Figure 4 presents the pooled adjusted RR for the association of diabetes and bladder cancer stratified by gender. According to the results, diabetes increased the risk of bladder cancer in women (RR, 1.23; 95% CI, 1.12 to 1.34) and in men (RR, 1.19; 95% CI, 1.09 to 1.30). There was significant heterogeneity among women (I^2^=56.5%; p=0.014) and men (I^2^=84.5%; p<0.001), but heterogeneity was lower among women. A sensitivity analysis showed that no single study was a potential source of heterogeneity. We determined the possibility of publication bias using a funnel plot (Figure 5), as well as the Begg and Egger tests. The studies were almost symmetrically scattered on both sides of the vertical line, showing the absence of publication bias. The Begg (p=0.646) and Egger (p=0.181) tests also showed no evidence of publication bias.

### Excessive body weight and bladder cancer risk

**Figure 6.**
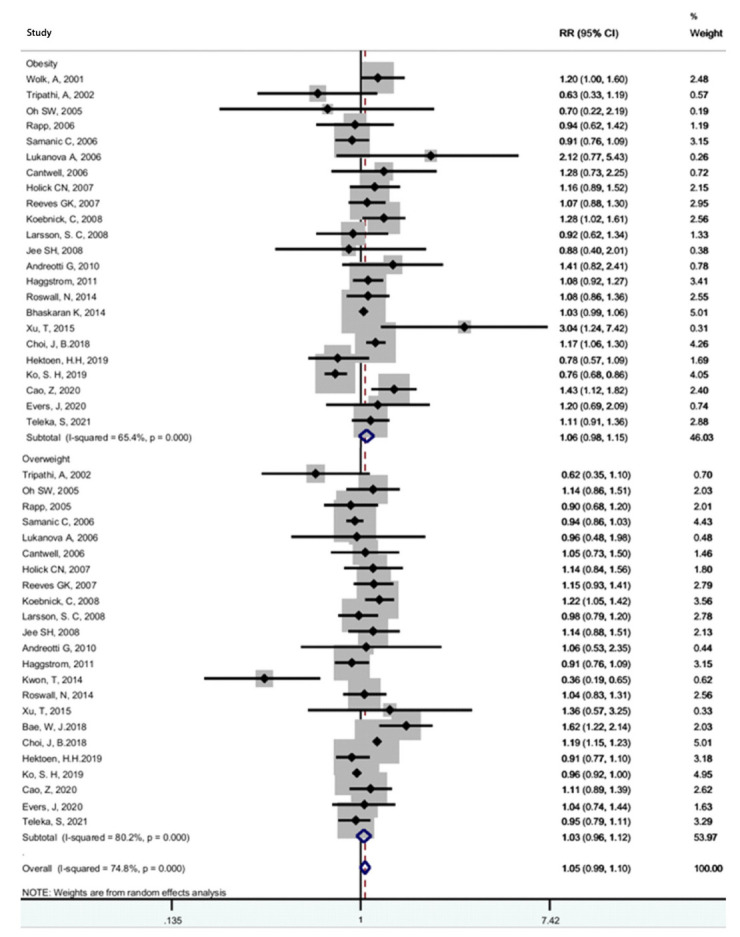
Forest plot of the association between overweight (BMI ≥25 kg/m^2^) or obesity (BMI ≥30 kg/m^2^) and bladder cancer^1^. RR, relative risk; CI, confidence interval; BMI, body mass index. ^1^Tumors or cancer of the urinary bladder.

The results of the relationship between excessive body weight and bladder cancer stratified by obesity and overweight are shown in Figure 6. Twenty-five studies were included [19,21,23,28,32,33,35-37,39,40,42-44,47-51,54,63,70,73,74,77]. With respect to excessive body weight status, no associations between obesity (RR, 1.06; 95% CI, 0.98 to 1.15) or overweight (RR, 1.03; 95% CI, 0.96 to 1.12) and bladder cancer were found. Significant heterogeneity was observed in the obesity (I^2^=65.4%; p<0.001) and overweight (I^2^=80.2%; p<0.001) groups. Sensitivity analysis showed that the studies by Choi et al. [43] and Ko et al. [47] were considerable sources of the observed heterogeneity. The Begg (p=0.484) and Egger (p=0.684) tests showed no evidence of publication bias.

### Hypertension and bladder cancer risk

**Figure 7.**
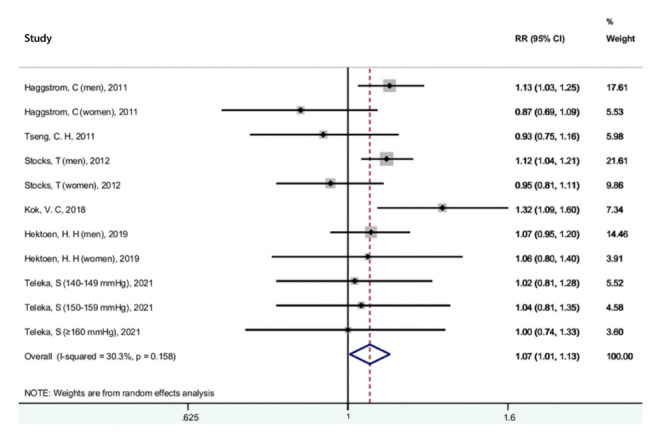
Forest plot of the association between hypertension and bladder cancer. RR, relative risk; CI, confidence interval.

The results for the relationship between hypertension and bladder cancer are shown in Figure 7. Six studies were included [19,37,38,56,60,73]. A statistically significant association was found between hypertension and bladder cancer (RR, 1.07; 95% CI, 1.01 to 1.13). There was no evidence of heterogeneity among these studies (I^2^=30.3%; p=0.158). A sensitivity analysis found that no single study significantly changed the pooled RR, and the Begg (p=0.312) and Egger (p=0.139) tests showed no evidence of publication bias.

## DISCUSSION

In the present study, PubMed, Web of Science, Embase, and Scopus were searched and 55 studies were identified. Our analysis demonstrated a statistically significant relationship between metabolic syndrome and bladder cancer, and there was moderate heterogeneity among these studies. Our findings also indicated statistically significant relationships between diabetes and hypertension and bladder cancer, but no association was found between obesity or overweight and bladder cancer.

Previous studies have reported metabolic syndrome as an essential factor for the progression of different types of cancers [78,79]. In developed countries worldwide, bladder cancer is one of most prevalent cancers, and it has been reported to show associations with metabolic syndrome risk factors including obesity, body mass index (BMI), blood pressure (BP) [80,81]. The results of our study were similar to those of a previous meta-analysis that found a statistically significant association between metabolic syndrome and the risk of bladder cancer [24]. The result of the meta-analysis conducted by Esposito et al. [78] showed a statistically significant relationship between metabolic syndrome and risk of bladder cancer in men, but not in women. Furthermore, this study focus specifically on the metabolic syndrome subgroup (not each subgroup), since it is this subgroup for which few studies were found and a separate analysis for men and women was not reported (in contrast, for example, to the subgroup of studies on diabetes, which contained many more studies).

In our study, there was a statistically significant association between diabetes and the risk of bladder cancer in both genders. The previous meta-analysis also found that diabetes increased the risk of bladder cancer [82]. It has been proven that insulin resistance and hyperinsulinemia stimulate tumor growth [83] and increase the risk of urinary tract infections [82], which are associated with the risk of bladder cancer [84]. Furthermore, diabetes changes the composition of urine and bladder function, which can increase the concentration or duration of exposure to carcinogens in the urine. This may also increase the risk of bladder cancer [85-87].

In the present meta-analysis, we observed no association between obesity or overweight and bladder cancer. Some studies have similarly found no association between overweight or obesity and bladder cancer [32,51,74], but others have found positive associations [42,43,48]. Obesity is characterized by an increase in adipose tissue and is often associated with worsening of the components of metabolic syndrome, such as hypertension, dyslipidemia, and insulin resistance. Obesity is associated with an increased risk of non-communicable diseases, such as cancer [88]. However, the relationship between obesity and the risk of bladder cancer remains speculative.

We found a statistically significant relationship between hypertension and bladder cancer. The results of previous studies similarly showed an association between hypertension and urinary bladder cancer [19,89,90]. A recent nationwide population-based cohort study supported a positive association between hypertension and the subsequent development of urinary bladder cancer [60]. Hypertension, as a global public health problem, is associated with multiple medical conditions [91-93]. Animal studies have provided evidence indicating that oxidative stress might play a causative role in the pathogenesis of hypertension [94], and oxidative stress is a risk factor in the development of cancer.

We used the Q-test and the I^2^ statistic to detect heterogeneity. Moderate heterogeneity was found for the relationship between metabolic syndrome and the risk of bladder cancer among studies. Regarding the components of metabolic syndrome, there was also significant heterogeneity in the relationship between diabetes and bladder cancer in both genders, and between obesity or overweight and bladder cancer. There can be various reasons for heterogeneity between studies. Most likely, this heterogenicity was due to the variety of definitions of metabolic syndrome, as well as because metabolic factors may not have been directly measured by the same method. For example, studies diagnosed high BP through direct BP measurements, self-reported diagnoses of hypertension, or specific drug use. This might have resulted in high heterogeneity between studies. Other relevant sources of heterogeneity may have been differences among studies in the sample size, publication date (the eligible studies were published from 2000 to 2021), geographical area, and the study population.

### Strengths and limitations

A strength of this study is that we included the most recent studies in the last 5 years. The second strength of this study is that we only assessed cohort studies in this systematic review, because cohort studies provide reliable and unbiased evidence. The third strength is that we evaluated all articles accurately to ensure that all articles were of sufficient quality for inclusion (fortunately, no articles were excluded due to quality issues). Finally, we performed subgroup analyses of diabetes, BMI, and BP, which are the most important components of metabolic syndrome, and we separately measured the effect of each of these factors on bladder cancer risk.

One of the limitations of the study is that we used studies written in the English language because non-English language studies were unavailable. The second limitation is that the studies we reviewed were from 2000 to 2021, and we did not include articles published before 2000. The reason for this is that the number of these articles was very limited and these articles did not meet our inclusion criteria.

## CONCLUSION

Our analysis demonstrated a statistically significant relationship between metabolic syndrome and the risk of bladder cancer. Of the components of metabolic syndrome, diabetes and hypertension showed statistically significant relationships with the risk of bladder cancer, but there was no association between obesity or overweight and the risk of bladder cancer.

## SUPPLEMENTARY MATERIALS

Supplementary Material 1. Assessment of Study Quality included in the meta-analysis

Supplementary Material 2. Characteristics of included studies in the meta-analysis
