# Supplementary material for: The relationship between metabolic syndrome and its components with bladder cancer: a systematic review and meta-analysis of cohort studies
Source: Epidemiol Health. 2022 May 30;44:e2022050. doi: 10.4178/epih.e2022050 (PMC9684010; doi:10.4178/epih.e2022050)
Supplement: Supplementary Material 1. — Assessment of Study Quality included in the meta-analysis [file epih-44-e2022050-suppl1.docx]

Supplementary Material 1. Assessment of Study Quality included in the meta-analysis

| study | Selection | | | | Comparability | Outcome | | | Total score |
| --- | --- | --- | --- | --- | --- | --- | --- | --- | --- |
|  | Representativeness of the exposed cohort | Selection of the non-exposed cohort | Ascertainment of exposure | Outcome of interest was not present at start of study | Comparability of cohorts on the basis of the design or analysis | Assessment of outcome | Was follow-up long enough for outcomes to occur | Adequacy of follow up of cohorts |  |
| Andreotti G, 2010 | 0 | 1 | 0 | 1 | 1 | 1 | 1 | 0 | 5 |
| Atchison EA, 2011 | 1 | 1 | 1 | 1 | 0 | 1 | 1 | 0 | 6 |
| Bae, W. J.2018 | 1 | 1 | 1 | 1 | 1 | 1 | 1 | 0 | 7 |
| Ballotari, p. 2017 | 1 | 1 | 1 | 1 | 1 | 1 | 1 | 1 | 8 |
| Bhaskaran, K.2014 | 1 | 1 | 1 | 1 | 1 | 1 | 1 | 0 | 7 |
| Cao, Z. 2020 | 1 | 1 | 1 | 1 | 1 | 1 | 1 | 1 | 8 |
| Cantwell, M. M. 2006 | 1 | 1 | 0 | 0 | 1 | 1 | 1 | 1 | 6 |
| Chen,HF 2015 | 1 | 1 | 1 | 1 | 0 | 1 | 1 | 0 | 6 |
| Choi, J. B. 2018 | 1 | 1 | 1 | 1 | 1 | 1 | 1 | 0 | 7 |
| Colmers IN 2013 | 1 | 1 | 1 | 0 | 1 | 1 | 1 | 1 | 7 |
| Evers, J. 2020 | 1 | 1 | 0 | 0 | 1 | 1 | 1 | 0 | 5 |
| Goossens, M. E. 2015 | 1 | 1 | 1 | 0 | 1 | 1 | 1 | 1 | 7 |
| Häggström, C.2011 | 1 | 1 | 1 | 1 | 1 | 1 | 1 | 0 | 7 |
| Hektoen, H. H. 2019 | 1 | 1 | 1 | 0 | 1 | 1 | 1 | 1 | 7 |
| Hemminki, K. 2010 | 1 | 1 | 1 | 0 | 0 | 1 | 1 | 1 | 6 |
| Holick CN, 2007 | 0 | 1 | 0 | 1 | 1 | 0 | 1 | 1 | 5 |
| Huang, W. L. 2020 | 0 | 1 | 1 | 0 | 0 | 1 | 1 | 1 | 5 |
| Inoue, M, 2006 | 1 | 1 | 0 | 1 | 1 | 1 | 1 | 0 | 6 |
| Jee,SH. 2005 | 1 | 1 | 1 | 1 | 1 | 1 | 1 | 1 | 8 |
| Khan M, 2006 | 1 | 1 | 1 | 1 | 1 | 1 | 1 | 0 | 7 |
| Kim, J. W. 2020 | 1 | 1 | 1 | 1 | 1 | 1 | 1 | 0 | 7 |
| Kim, S. K. 2020 | 1 | 1 | 0 | 1 | 1 | 1 | 1 | 0 | 6 |
| Koebnick, C. 2008 | 1 | 1 | 0 | 1 | 1 | 1 | 1 | 1 | 7 |
| Ko, S. H. 2019 | 1 | 1 | 1 | 0 | 1 | 1 | 1 | 1 | 7 |
| Kok, V. C. 2018 | 1 | 1 | 1 | 1 | 0 | 1 | 1 | 1 | 7 |
| Kwon, T. 2014 | 0 | 1 | 1 | 1 | 0 | 1 | 1 | 1 | 6 |
| Lai, G. Y. 2013 | 1 | 1 | 0 | 1 | 1 | 1 | 1 | 0 | 6 |
| Larsson, S. C. 2008 | 1 | 0 | 0 | 1 | 1 | 1 | 1 | 1 | 6 |
| Lee, H. Y. 2020 | 1 | 1 | 0 | 0 | 1 | 1 | 1 | 1 | 6 |
| Lin, C. C. 2014 | 1 | 1 | 1 | 1 | 1 | 1 | 1 | 0 | 7 |
| Lo, S. F. 2013 | 1 | 1 | 1 | 1 | 1 | 1 | 1 | 0 | 7 |
| Lukanova A, 2006 | 1 | 1 | 0 | 1 | 1 | 1 | 1 | 1 | 7 |
| Newton, C. C.2013 | 1 | 1 | 0 | 0 | 1 | 1 | 1 | 1 | 6 |
| Ogunleye, A. A. 2009 | 1 | 1 | 1 | 0 | 1 | 1 | 1 | 1 | 7 |
| Oh SW, 2005 | 0 | 1 | 1 | 1 | 1 | 1 | 1 | 1 | 7 |
| Peila, R. 2020 | 1 | 1 | 1 | 1 | 1 | 1 | 1 | 1 | 8 |
| Prizment, A. E. 2013 | 1 | 1 | 0 | 1 | 1 | 1 | 1 | 0 | 6 |
| Rastad, H. 2019 | 1 | 1 | 1 | 1 | 1 | 1 | 1 | 1 | 1 |
| Roswall, N. 2014 | 1 | 1 | 1 | 1 | 1 | 1 | 1 | 1 | 8 |
| Rapp,K .2005 | 1 | 1 | 1 | 1 | 1 | 1 | 1 | 1 | 8 |
| Rapp, K. 2006 | 1 | 1 | 1 | 1 | 1 | 1 | 1 | 1 | 8 |
| Reeves GK, 2007 | 1 | 1 | 0 | 1 | 1 | 1 | 1 | 0 | 6 |
| Russo, A. 2008 | 1 | 1 | 1 | 1 | 0 | 1 | 1 | 0 | 6 |
| Samanic C, 2006 | 1 | 1 | 1 | 1 | 1 | 1 | 1 | 1 | 8 |
| Stocks, T. 2012 | 1 | 1 | 1 | 1 | 1 | 1 | 1 | 0 | 7 |
| Swerdlow, A. J 2005 | 1 | 1 | 1 | 1 | 1 | 1 | 1 | 1 | 8 |
| Teleka, S. 2021 | 1 | 1 | 1 | 0 | 1 | 1 | 1 | 1 | 7 |
| Tseng, C. H. 2011 | 1 | 1 | 1 | 1 | 1 | 1 | 1 | 0 | 7 |
| Tripathi, A .2002 | 1 | 1 | 0 | 1 | 1 | 1 | 1 | 1 | 7 |
| Walker, J. J. 2013 | 0 | 1 | 1 | 0 | 0 | 1 | 1 | 1 | 5 |
| Wolk, A .2001 | 1 | 1 | 1 | 0 | 0 | 1 | 1 | 0 | 5 |
| Woolcott, C. G. 2011 | 1 | 1 | 0 | 1 | 1 | 1 | 1 | 0 | 6 |
| Xu HL, 2015 | 1 | 1 | 1 | 0 | 1 | 1 | 1 | 0 | 6 |
| Xu, T. 2015 | 1 | 1 | 1 | 1 | 1 | 1 | 1 | 0 | 7 |
| Yood,M,U, 2009 | 1 | 1 | 1 | 1 | 1 | 1 | 1 | 0 | 7 |

1. Wolk A, Gridley G, Svensson M, Nyrén O, McLaughlin JK, Fraumeni JF, et al. A prospective study of obesity and cancer risk (Sweden). Cancer causes & control. 2001;12(1):13-21.

2. Tripathi A, Folsom AR, Anderson KE. Risk factors for urinary bladder carcinoma in postmenopausal women: the Iowa Women's Health Study. Cancer: Interdisciplinary International Journal of the American Cancer Society. 2002;95(11):2316-23.

3. Jee SH, Ohrr H, Sull JW, Yun JE, Ji M, Samet JM. Fasting serum glucose level and cancer risk in Korean men and women. Jama. 2005;293(2):194-202.

4. Swerdlow AJ, Laing SP, Qiao Z, Slater SD, Burden AC, Botha JL, et al. Cancer incidence and mortality in patients with insulin-treated diabetes: a UK cohort study. British Journal of Cancer. 2005;92(11):2070-5.

5. Rapp K, Schroeder J, Klenk J, Stoehr S, Ulmer H, Concin H, et al. Obesity and incidence of cancer: a large cohort study of over 145 000 adults in Austria. British journal of cancer. 2005;93(9):1062-7.

6. Oh SW, Yoon YS, Shin S-A. Effects of excess weight on cancer incidences depending on cancer sites and histologic findings among men: Korea National Health Insurance Corporation Study. Journal of Clinical Oncology. 2005;23(21):4742-54.

7. Inoue M, Iwasaki M, Otani T, Sasazuki S, Noda M, Tsugane S, et al. Diabetes mellitus and the risk of cancer: results from a large-scale population-based cohort study in Japan. Archives of internal medicine. 2006;166(17):1871-7.

8. Cantwell MM, Lacey Jr JV, Schairer C, Schatzkin A, Michaud DS. Reproductive factors, exogenous hormone use and bladder cancer risk in a prospective study. International journal of cancer. 2006;119(10):2398-401.

9. Samanic C, Chow W-H, Gridley G, Jarvholm B, Fraumeni JF. Relation of body mass index to cancer risk in 362,552 Swedish men. Cancer Causes & Control. 2006;17(7):901-9.

10. Lukanova A, Björ O, Kaaks R, Lenner P, Lindahl B, Hallmans G, et al. Body mass index and cancer: results from the Northern Sweden Health and Disease Cohort. International journal of cancer. 2006;118(2):458-66.

11. Rapp K, Schroeder J, Klenk J, Ulmer H, Concin H, Diem G, et al. Fasting blood glucose and cancer risk in a cohort of more than 140,000 adults in Austria. Diabetologia. 2006;49(5):945-52.

12. Khan M, Mori M, Fujino Y, Shibata A, Sakauchi F, Washio M, et al. Site-specific cancer risk due to diabetes mellitus history: evidence from the Japan Collaborative Cohort (JACC) Study. Asian Pacific Journal of Cancer Prevention. 2006;7(2):253-9.

13. Holick CN, Giovannucci EL, Stampfer MJ, Michaud DS. Prospective study of body mass index, height, physical activity and incidence of bladder cancer in US men and women. International journal of cancer. 2007;120(1):140-6.

14. Reeves GK, Pirie K, Beral V, Green J, Spencer E, Bull D. Cancer incidence and mortality in relation to body mass index in the Million Women Study: cohort study. Bmj. 2007;335(7630):1134.

15. Koebnick C, Michaud D, Moore SC, Park Y, Hollenbeck A, Ballard-Barbash R, et al. Body mass index, physical activity, and bladder cancer in a large prospective study. Cancer Epidemiology Biomarkers and Prevention. 2008;17(5):1214-21.

16. Larsson SC, Andersson S-O, Johansson J-E, Wolk A. Diabetes mellitus, body size and bladder cancer risk in a prospective study of Swedish men. European Journal of Cancer. 2008;44(17):2655-60.

17. Russo A, Autelitano M, Bisanti L. Metabolic syndrome and cancer risk. European Journal of Cancer. 2008;44(2):293-7.

18. Jee SH, Yun JE, Park EJ, Cho ER, Park IS, Sull JW, et al. Body mass index and cancer risk in Korean men and women. International journal of cancer. 2008;123(8):1892-6.

19. Yood MU, Oliveria SA, Campbell UB, Koro CE. Incidence of cancer in a population-based cohort of patients with type 2 diabetes. Diabetes & Metabolic Syndrome: Clinical Research & Reviews. 2009;3(1):12-6.

20. Ogunleye AA, Ogston SA, Morris AD, Evans J. A cohort study of the risk of cancer associated with type 2 diabetes. British journal of cancer. 2009;101(7):1199-201.

21. Andreotti G, Hou L, Freeman LEB, Mahajan R, Koutros S, Coble J, et al. Body mass index, agricultural pesticide use, and cancer incidence in the Agricultural Health Study cohort. Cancer Causes & Control. 2010;21(11):1759-75.

22. Hemminki K, Li X, Sundquist J, Sundquist K. Risk of cancer following hospitalization for type 2 diabetes. The Oncologist. 2010;15(6):548.

23. Häggström C, Stocks T, Rapp K, Bjørge T, Lindkvist B, Concin H, et al. Metabolic syndrome and risk of bladder cancer: prospective cohort study in the metabolic syndrome and cancer project (Me‐Can). International journal of cancer. 2011;128(8):1890-8.

24. Atchison EA, Gridley G, Carreon JD, Leitzmann MF, McGlynn KA. Risk of cancer in a large cohort of US veterans with diabetes. International journal of cancer. 2011;128(3):635-43.

25. Tseng CH. Diabetes and risk of bladder cancer: A study using the National Health Insurance database in Taiwan. Diabetologia. 2011;54(8):2009-15.

26. Woolcott CG, Maskarinec G, Haiman CA, Henderson BE, Kolonel LN. Diabetes and urothelial cancer risk: The Multiethnic Cohort Study. Cancer Epidemiology. 2011;35(6):551-4.

27. Stocks T, Van Hemelrijck M, Manjer J, Bjørge T, Ulmer H, Hallmans G, et al. Blood pressure and risk of cancer incidence and mortality in the metabolic syndrome and cancer project. Hypertension. 2012;59(4):802-10.

28. Lai GY, Park Y, Hartge P, Hollenbeck AR, Freedman ND. The association between self-reported diabetes and cancer incidence in the NIH-AARP Diet and Health Study. The Journal of Clinical Endocrinology & Metabolism. 2013;98(3):E497-E502.

29. Lo SF, Chang SN, Muo CH, Chen SY, Liao FY, Dee SW, et al. Modest increase in risk of specific types of cancer types in type 2 diabetes mellitus patients. International Journal of Cancer. 2013;132(1):182-8.

30. Newton CC, Gapstur SM, Campbell PT, Jacobs EJ. Type 2 diabetes mellitus, insulin-use and risk of bladder cancer in a large cohort study. International Journal of Cancer. 2013;132(9):2186-91.

31. Walker JJ, Brewster DH, Colhoun HM, Fischbacher CM, Leese GP, Lindsay RS, et al. Type 2 diabetes, socioeconomic status and risk of cancer in Scotland 2001-2007. Diabetologia. 2013;56(8):1712-5.

32. Prizment AE, Anderson KE, Yuan J-M, Folsom AR. Diabetes and risk of bladder cancer among postmenopausal women in the Iowa Women’s Health Study. Cancer Causes & Control. 2013;24(3):603-8.

33. Colmers IN, Majumdar SR, Yasui Y, Bowker SL, Marra CA, Johnson JA. Detection bias and overestimation of bladder cancer risk in type 2 diabetes: a matched cohort study. Diabetes care. 2013;36(10):3070-5.

34. Bhaskaran K, Douglas I, Forbes H, dos-Santos-Silva I, Leon DA, Smeeth L. Body-mass index and risk of 22 specific cancers: a population-based cohort study of 5· 24 million UK adults. The Lancet. 2014;384(9945):755-65.

35. Kwon T, Jeong IG, You D, Han KS, Hong S, Hong B, et al. Obesity and prognosis in muscle-invasive bladder cancer: The continuing controversy. International Journal of Urology. 2014.

36. Lin CC, Chiang JH, Li CI, Liu CS, Lin WY, Hsieh TF, et al. Cancer risks among patients with type 2 diabetes: A 10-year follow-up study of a nationwide population-based cohort in Taiwan. BMC Cancer. 2014;14(1).

37. Roswall N, Freisling H, Bueno‐de‐Mesquita H, Ros M, Christensen J, Overvad K, et al. Anthropometric measures and bladder cancer risk: a prospective study in the EPIC cohort. International journal of cancer. 2014;135(12):2918-29.

38. Goossens ME, Zeegers MP, Bazelier MT, De Bruin ML, Buntinx F, De Vries F. Risk of bladder cancer in patients with diabetes: A retrospective cohort study. BMJ Open. 2015;5(6).

39. Chen H-F, Chen S-W, Chang Y-H, Li C-Y. Risk of malignant neoplasms of kidney and bladder in a cohort study of the diabetic population in Taiwan with age, sex, and geographic area stratifications. Medicine. 2015;94(38).

40. Xu H-L, Fang H, Xu W-H, Qin G-Y, Yan Y-J, Yao B-D, et al. Cancer incidence in patients with type 2 diabetes mellitus: a population-based cohort study in Shanghai. BMC cancer. 2015;15(1):1-8.

41. Xu T, Zhu Z, Wang X, Xia L, Zhang X, Zhong S, et al. Impact of body mass on recurrence and progression in Chinese patients with Ta, T1 urothelial bladder cancer. International Urology and Nephrology. 2015;47(7):1135-41.

42. Ballotari P, Vicentini M, Manicardi V, Gallo M, Chiatamone Ranieri S, Greci M, et al. Diabetes and risk of cancer incidence: Results from a population-based cohort study in northern Italy. BMC Cancer. 2017;17(1).

43. Bae WJ, Choi JB, Moon HW, Park YH, Cho HJ, Hong SH, et al. Influence of diabetes on the risk of urothelial cancer according to body mass index: A 10-year nationwide population-based observational study. Journal of Cancer. 2018;9(3):488-93.

44. Kok VC, Zhang HW, Lin CT, Huang SC, Wu MF. Positive association between hypertension and urinary bladder cancer: epidemiologic evidence involving 79,236 propensity score-matched individuals. Upsala journal of medical sciences. 2018;123(2):109-15.

45. Choi JB, Lee EJ, Han KD, Hong SH, Ha US. Estimating the impact of body mass index on bladder cancer risk: Stratification by smoking status. Scientific reports. 2018;8(1):947.

46. Hektoen HH, Stenehjem JS, Robsahm TE, Andreassen BK, Gislefoss R. Associations between lifestyle factors and risk of bladder cancer, in a large population-based Norwegian cohort. Norsk Epidemiologi. 2019;28:70.

47. Ko SH, Han KD, Yun JS, Chung S, Koh ES. Impact of obesity and diabetes on the incidence of kidney and bladder cancers: A nationwide cohort study. European Journal of Endocrinology. 2019;181(5):489-98.

48. Rastad H, Parsaeian M, Shirzad N, Mansournia MA, Yazdani K. Diabetes mellitus and cancer incidence: the Atherosclerosis Risk in Communities (ARIC) cohort study. Journal of Diabetes and Metabolic Disorders. 2019;18(1):65-72.

49. Cao Z, Zheng X, Yang H, Li S, Xu F, Yang X, et al. Association of obesity status and metabolic syndrome with site-specific cancers: a population-based cohort study. British Journal of Cancer. 2020;123(8):1336-44.

50. Evers J, Grotenhuis AJ, Aben KKH, Kiemeney L, Vrieling A. No clear associations of adult BMI and diabetes mellitus with non-muscle invasive bladder cancer recurrence and progression. PLoS One. 2020;15(3):e0229384.

51. Huang WL, Huang KH, Huang CY, Pu YS, Chang HC, Chow PM. Effect of diabetes mellitus and glycemic control on the prognosis of non-muscle invasive bladder cancer: a retrospective study. BMC Urology. 2020;20(1).

52. Kim JW, Ahn ST, Oh MM, Moon DG, Cheon J, Han K, et al. Increased incidence of bladder cancer with metabolically unhealthy status: analysis from the National Health Checkup database in Korea. Scientific reports. 2020;10(1):6476.

53. Kim SK, Jang JY, Kim DL, Rhyu YA, Lee SE, Ko SH, et al. Site-specific cancer risk in patients with type 2 diabetes: A nationwide population-based cohort study in Korea. Korean Journal of Internal Medicine. 2020;35(3):641-51.

54. Lee HY, Tang JH, Chen YH, Wu WJ, Juan YS, Li WM, et al. The metabolic syndrome is associated with the risk of urothelial carcinoma from a health examination database. International Journal of Clinical Oncology. 2020.

55. Peila R, Rohan TE. Diabetes, glycated hemoglobin, and risk of cancer in the UK Biobank Study. Cancer Epidemiology Biomarkers and Prevention. 2020;29(6):1107-19.

56. Teleka S, Jochems SHJ, Häggström C, Wood AM, Järvholm B, Orho-Melander M, et al. Association between blood pressure and BMI with bladder cancer risk and mortality in 340,000 men in three Swedish cohorts. Cancer Medicine. 2021;10(4):1431-8.
