# Supplementary material for: The relationship between metabolic syndrome and its components with bladder cancer: a systematic review and meta-analysis of cohort studies
Source: Epidemiol Health. 2022 May 30;44:e2022050. doi: 10.4178/epih.e2022050 (PMC9684010; doi:10.4178/epih.e2022050)
Supplement: Supplementary Material 2. — Characteristics of included studies in the meta-analysis [file epih-44-e2022050-suppl2.docx]

Supplementary Material 2. Characteristics of included studies in the meta-analysis

| Author/year | country | Study purpose | Sample characteristics | Sample size | Exposure | Mean  Rang\ age | sex | confounding | Main momentary measurements | components of metabolic syndrome definition | |
| --- | --- | --- | --- | --- | --- | --- | --- | --- | --- | --- | --- |
| Andreotti G, 2010 | USA | Obesity is associated with increased risks of several cancers | Using data from the Agricultural Health Study. Pesticide applicators residing in Iowa and North Carolina and their spouses were enrolled between 1993 and 1997 and given a self-administered questionnaire to obtain pesticide use and other information | 57,310 | BMI | 40-70< | both | smoking status, hypertension, taking vitamin supplements, parity, race, vegetable consumption, exercise, pack-years of cigarette smoking | Overweight RR= 1.06, 95% CL= 0.53, 2.35). | overweight (25–29.9 kg/m2), obese I (30.0–34.9 kg/m2) and obese II/III (≥35 kg/m2) | |
| Atchison EA,  2011 | USA | assess the risk of cancer among diabetic men | The study cohort was identified using hospital discharge records dated between July 1, 1969 and September 30, 1996 from 142 nationwide United States Veterans Affairs (VA) hospitals. | 4,501,578 | Diabetes | 4-79 | men | age, time, latency, race and number of visits and diagnoses of alcohol-related conditions, obesity and chronic obstructive pulmonary disease. | RR=0.96, (95%CI=0.92-1.01) | NA | |
| Bae, W. J.2018 | Korea | association between obesity and urothelial cancer | (NHIS) contains an extensive information about the health problem of most Korean population.  This study developed a customized database. Using NHIS, we generated a sample and selected by a systematic sampling method to generate a representative sample from the total Korean population existing in 2004 | 1,025,340 | BMI | aged >20 |  | age, hypertension, dyslipidemia, smoking status, alcohol consumption, exercise | Overweight HR=1.62, 95% CI= 1.22, 2.14 | normal weight (18.5 to 22.9); overweight (23 to 24.9); and obese (over 25) | |
| Ballotari, p.  2017 | Italy | compare cancer incidence in populations with and without diabetes by cancer site | By use of the Reggio Emilia diabetes registry, we classified the resident population December 31^st^ 2009 into two groups: with and without diabetes. By linking with the cancer registry, we calculated the 2010-2013 cancer incidence in both groups | 407157 | Diabetes | 20-84 | both | age, foreign status, sex | IRR = 1.39, 95%CI= 1.16, 1.68 | NA | |
| Bhaskaran, K.2014 | UK | BMI and the most common site-specific cancers | collected data from the UK Clinical Practice Research Datalink (CPRD) | 5 243 978 | BMI | 27-57 | both | age, smoking status, alcohol use, previous diabetes diagnosis, index of multiple deprivation, calendar period, sex | HR=1.03, 95% CI=.99, 1.06 | BMI (overweight ≥ 25 kg/m2, obesity ≥ 30 kg/m2) | |
| Cao, Z.  2020 | UK | Association of obesity status and metabolic syndrome with site-specific cancers | used data derived from individuals from the UK Biobank who were enrolled from 2006-2016 | 390,575 | BMI | 37-73 | both | sex, age, ethnicity, Townsend deprivation index, qualification,  employment status, alcohol in taking, smoking status | overweight HR=1.11, 95%CL=.89, 1.39 obese HR=1.43, 95%CL=1.12, 182 | BMI (overweight ≥ 25 kg/m2, obesity ≥ 30 kg/m2) | |
| Cantwell, M. M.  2006 | USA | reproductive factors, oral contraceptive use and postmenopausal hormone use, and risk of bladder cancer | National Cancer Institute (NCI) | 54،308 | BMI | 50-75 | women | age, calendar year,  smoking status | Overweight HR=1.05, 95% CL= 0.73, 1.50  Obesity HR=1.28, 95% CL= 0.73, 2.25 | BMI (overweight ≥ 25 kg/m2, obesity ≥ 30 kg/m2) | |
| Chen,HF 2015 | Taiwan | Incidence and relative risks of kidney and bladder neoplasms in diabetic population of Taiwan. | All the patients who sought ambulatory cares for diabetes (International Classification of Diagnosis, 9th version, Clinical Modification [ICD-9-CM]: 250 or A-code: A181) in 2000 were recruited in the diabetic group.  The control group was randomly selected from the all NHI beneficiaries registered in 2000, | Diabetic patients (n = 615,532)  controls (n = 614,871) | Diabetes | 45-60< | both | age, sex, geographic area, urbanization status, frequency of medical visit | men HR= 1.13, 95% CI= 1.04, 1.23).  women HR= 1.04, 95% CI= .93, 1.16 all HR= 1.09, 95% CI= 1.02, 1.17 | NA | |
| Choi, J. B.  2018 | Korea | associations between obesity and bladder cancer risk have reported inconsistent results | Using nationally representative data from the National Health Insurance System (NHIS). Among people who underwent at least one health examination from 2009 to 2012 in Korea, men without a previous diagnosis of bladder cancer were followed up until December 2015. | 11,823,876 | BMI | 30-65 | Men, | age, diabetes, smoking status, exercise, alcohol consumption | overweight HR= 1.19, 95% CI= 1.15, 1.23  obesity HR= 1.17, 95% CI= 1.06, 1.3 | overweight (23–24.9); obese class 1 (25–29.9); and obese class 2 (over 30) | |
| Colmers IN 2013 | Canada | To investigate whether the risk of bladder cancer in individuals with newly diagnosed type 2 diabetes. | With the use of linked administrative databases from 1996 to 2006, we established a cohort of adults from British Columbia, Canada, | 185,100 | Diabetes | 55-69 | women | age, sex, socioeconomic status | diagnosed 3–10 years HR= 1.3 (95% CI= 1.02, 1.67  diagnosed <l year HR=1.05, 95% CI= 0.88, 1.24 | NA | |
| Evers, J.  2020 | Netherland | associations of BMI and diabetes mellitus with risks of recurrence and progression among non-muscle invasive bladder cancer patients | A population-based cohort of patients diagnosed with non-muscle invasive bladder cancer between 1995 and 2010 was retrospectively identified from the Netherlands Cancer Registry and invited to participate in the Nijmegen Bladder Cancer Study | 1,433 | BMI, Diabetes | 56.0- 70.0 | men | age at time of UBC diagnosis, gender, highest completed level of education, weekly duration of physical activity, history of urinary bladder cancer among first degree relatives, tumor stage, tumor grade, and presence of concomitant CIS | Overweight HR = 1.04, 95% CI= 0.74, 1.44  obesity HR = 1.20, 95% CI= 0.69, 2.09  Diabetes HR=1.16, 95% CI= 0.76, 1.76 | | BMI (overweight ≥ 25 kg/m2, obesity ≥ 30 kg/m2), |
| Goossens, M. E.  2015 | UK | Examine the association between diabetes, and both urinary bladder cancer (UBC) risk and mortality. | A retrospective cohort study using data from the UK Clinical Practice Research Data link (CPRD) linked to the Office of National Statistics (ONS). | 329 168 | Diabetes | 18-82< | both | age, sex, smoking, body mass index | ALL HR= 1.09, 95%CL= 0.97, 1.21  men HR= 1.11, 95%CL= 0.98, 1.25  women HR= 1.01, 95%CL= 0.79, 1.29 | | NA |
| Häggström, C.2011 | Sweden | association between factors in the metabolic syndrome (MetS) and risk of bladder cancer | In brief, the Me-Can project consists of seven different cohorts, from Norway; the Oslo study I cohort (Oslo), Norwegian Counties Study (NCS), Cohort of Norway (CONOR), Sweden; Västerbotten Intervention Project (VIP) and Malmö Preventive Project (MPP) and Austria; Vorarlberg Health Monitoring and Prevention Programme (VHM & PP) | 578,700 | Metabolic Syndrome (MetS), blood pressure, BMI | 40-55< | both | smoking, five categories of birth date, age at measurement | Men, Mets RR=1.10, 95% CI= 1.01, 1.18  Women, Mets RR=.96, 95% CI= .79, 1.184  Men, Blood pressure, RR= 1.13,  95% CI= 1.03,1.25. women, Blood pressure RR= .87, 95% CI= .69, 1.09.  Overweight RR=0.91, 95% CI 0.77, 1.10 | | BMI (overweight ≥ 25 kg/m2, obesity ≥ 30 kg/m2), systolic blood pressure (hypertension ≥ 140 mmHg), and diastolic blood pressure (hypertension ≥90 mmHg). impaired fasting glucose (6.1–6.9 mmol/l) and diabetes (≥ 7.0 mmol/l) among subjects who had fasted > 8 hours prior to blood draw |
| Hektoen, H. H.  2019 | Norway | examined relations between lifestyle associated factors (e.g., BMI, physical activity and metabolic disorders) and risk of bladder cancer, | The Janus Serum Bank Cohort (Janus Cohort) has been created as a population‐based biobank for prospective cancer studies, containing serum samples and data from health examinations, including measured anthropometry and questionnaire data from Norwegians | 292 851 | BMI, Systolic blood pressure | 15-89 | both | Smoking, occupational exposure | overweight HR= 0.91, 95% CI = 0.77, 1.1  obesity HR= 0.78, 95% CI =0.57-1.09  systolic blood pressure in men HR =1.07 95% CI = 0.95, 1.2 in women HR =1.06 95% CI= 0.8, 1.4 | | underweight (<18.5 kg/m2), normal weight (18.5‐24.9 kg/m2) overweight (25‐29.9 kg/m2), and obese (≥30 kg/m2).  hypertension (SBP ≥ 140),(DBP ≥ 90) |
| Hemminki, K. 2010 | Sweden | determining subsequent cancer risks in patients hospitalized for T2D | T2D patients were obtained from the nationwide Hospital Discharge Register; cancers were recorded from the Swedish Cancer Registry | 125126 | Diabetes | 39< | both | NA | SIR=1.37, 95% CL= 1.25, 1.49 | | NA |
| Holick CN,  2007 | USA | association between body mass index (BMI), height, recreational physical activity and the risk of bladder cancer | Data were used from 2 ongoing cohorts, the Health Professionals Follow-up Study and the Nurses' Health Study, with 3,542,012 years of follow-up and 866 incident bladder cancer cases | 173229 | BMI | 30-75 | both | age, pack-years of cigarette smoking, current smoking. | Overweight RR= 1.14, 95% CL= 0.84, 1.56  Obese RR =1.16, 95% CL= 0.89, 1.52 | | normal weight (18.5-24.9 kg/m2), overweight (25.0-29.9 kg/m2), and obesity (≥30.0 kg/m2) |
| Huang, W. L.  2020 | Taiwan | Investigate the effect of DM and glycemic control on the prognosis of bladder cancer. | A retrospective chart review of a prospective database from January 2012 to December 2017. Patients with newly diagnosed non-muscle invasive bladder cancer (NMIBC) were included. | 287 | Diabetes | 67 | men | age, sex, history of smoking, BMI, hypertension, serum creatinine level, DM, glycemic control, metformin use, thiazolidinedione (TZD) use, clinical T1 stage, grade of urothelial carcinoma, concurrent CIS, tumor number, tumor size, intravesical therapy | OR = 0.73, 95% CI= 0.20, 2.60 | | NA |
| Inoue  M,  2006 | Japan | Association between diabetes mellitus (DM) and cancer. | This study in the Japan Public Health Center-Based Prospective Study A total general Japanese persons who responded to the baseline questionnaire, from January 1990 to December 1994, were followed up for cancer incidence through December 31, 2003. | 97771 | Diabetes | 40 - 69 | both | age, study area, history of cerebrovascular disease, history of ischemic heart  disease, smoking, ethanol intake, body mass index, leisure-time physical  activity, green vegetable intake, Coffee intake. | men HR= 1.63, 95% CL= 0.89, 3.00  women HR= 0.64, 95% CL= 0.09, 4.75 | | NA |
| Jee,SH  2005 | Korea | Relationship between fasting serum glucose and diabetes and risk of all cancers. | Ten-year prospective cohort study received health insurance from the National Health Insurance Corp and had a biennial medical evaluation in 1992-1995. | 1329525 | Diabetes | 30 - 95 | both | age, smoking, alcohol use. | RR=1.32, 95% CI 1.1, 1.57 | | NA |
| Khan M,  2006 | Japan | association of diabetes mellitus (DM) history with total and common site-specific cancers | This study using a large cohort of, extracted from healthy participants of the JACC Study who were aged 40-79 years and living in 24 municipalities in Japan. At enrollment during 1988-90, each subject completed a self-administered questionnaire | 56881 | Diabetes | 40-79 | men | age, BMI, smoking, and drinking | IRR=1.3, 95% CI= 0.41, 2.6 | | NA |
| Kim, J. W.  2020 | Korea | assessed the association between metabolic health status and the incidence of bladder cancer | This study using nationally representative data from the National Health Insurance System and National Health Checkups (NHC) databases in South Korea. Data for who participated in the NHC between 2009 and 2012 | 11,781,768 | MHO (metabolically healthy, obese) | 46.5  40-65< | men | age, smoking status, alcohol consumption, exercise, economic status | HR= 1.06, 95% CI= 1.01, 1.11 | | obese (BMI ≥ 25 kg/m2) and normal weight (BMI < 25 kg/m2).  triglyceride level ≥150 mg⁄dL, (HDL) cholesterol level <40 mg ⁄ dL, fasting glucose level ≥100 mg⁄ dL blood pressure (BP) ≥ 130⁄85 or waist circumference (WC) ≥ 90 cm. |
| Kim, S. K.  2020 | Korea | Aimed to evaluate site-specific cancer risk in diabetic patients and to investigate causal and temporal relationships by analyzing organ-specific cancer risk according to the duration of diabetes. | Using a database provided by the Korean National Health Insurance Service, we conducted a retrospective, population-based cohort study of adults aged ≥ 30 years from January 2005 to December 2013. | 25,709,497 | Diabetic | 47.7 | both | age, sex, income, place, hypertension, hyperlipidemia, chronic liver disease, ischemic heart disease, chronic kidney disease | All HR = 1.28, CL%95=1.23,1.33 men HR= 1.30, CL%95=1.24, 1.37 women HR= 1.23 CL%95=1.14, 1.32 | | NA |
| Koebnick, C.  2008 | USA | investigated the associations between BMI, physical activity, and bladder cancer | Use the NIH-AARP Diet and Health Study, a prospective cohort U.S. men and women, followed from 1995 to 2003. 1,199 ductal carcinoma cases, 739 lobular carcinoma cases, and 1,474 controls | 471,760 | BMI | 50 to 71 | both | age, gender, physical activity. | Overweight RR=1.22, 95% CI= 1.05, 1.42  Obese RR=1.28, 95% CI= 1.02, 1.61 | | normal weight (18.5-24.9 kg/m2), overweight (25.0-29.9 kg/m2), and obesity (≥30.0 kg/m2) |
| Ko, S. H.  2019 | Korea | Impact of obesity and diabetes on the incidence of kidney and bladder cancers | Using nationally representative data from the Korean National Health Insurance System, subjects without any malignancy who underwent health examinations in 2009 were followed to the end of 2017. | 9,777,133 | BMI | ≥20 | both | age, sex, smoking, drinking, exercise income, diabetes, hypertension and dyslipidemia | overweight HR= 0.96, 95% CI= 0.92, 1  obesity HR= 0.76, 95% CI= 0.68, 0.86 | | underweight (<18.5 kg/m2), normal weight (18.5–22.9 kg/m2), overweight (23–24.9 kg/m2), obese (25–29.9 kg/m2), and morbidly obese (>30 kg/m2) |
| Kok, V. C.  2018 | Taiwan | Hypothesized that hypertensive patients harbor a higher risk of urinary bladder (UB) cancer. | Study on adults using a National Health Insurance Research Database (NHIRD) dataset. all patients were completely followed up till the occurrence of incident UB cancer or death or till the last day of 2013 | 39,618 | Hypertension | 56 | both | Medical comorbidities of smoking-related diagnoses, morbid obesity, chronic liver disease. | Hypertension HR=1.32, 95% CI=1.09, 1.60 | | NA |
| Kwon, T.  2014 | Korea | To investigate the association between body mass index and clinic pathological features of bladder cancer | The medical records of 746 consecutive patients who underwent radical cystectomy for bladder cancer between August 1990 and March 2012 were retrospectively reviewed. | 714 | BMI | 62.4 | both | age, tumor  stage, histological grade, lymph vascular invasion, concomitant CIS, lymph node metastasis | Overweight HR= 0.36, 95% CI= 0.19, 0.65 | | normal (BMI <23 kg/m2), overweight (BMI 23–25 kg/m2) and obese (BMI ≥25.0 kg/m2) |
| Lai, G. Y. 2013 | USA | association between self-reported diabetes and cancer incidence | The NIH-AARP Diet and Health Study is a prospective cohort of men and women, in the United States who live in 6 states (California, Florida, Louisiana, New Jersey, North Carolina, and Pennsylvania) and 2 metropolitan areas (Atlanta, Georgia, and Detroit, Michigan) | 494867 | Diabetes | 50–71 | both | age, sex, BMI | HR=1.1, 95% CL= 1, 1.21 | | NA |
| Larsson, S. C.  2008 | Sweden | associations between a history of diabetes, height, weight, body mass index the incidence of bladder cancer | Cohort of Swedish Men, a prospective study of men. During follow-up from 1998 through December 2007 | 48,850 | Diabetes, BMI | 45–79 | men | age, education, smoking status, pack-years of  smoking | Overweight RR= 0.98, 95% CL= 0.79, 1.20  Obese RR= 0.92, 95% CL= 0.62, 1.34  diabetes RR= 1.16, 95% CL= 0.81, 1.64 | | overweight (25.0–29.9 kg/m2  ) and obesity (P30.0 kg/  m2 |
| Lee, H. Y.  2020 | Taiwan | Relationship between metabolic syndromes and urothelial carcinoma including urinary bladder urothelial carcinoma (UBUC) and upper tract urothelial carcinoma (UTUC). | Population-based cohort study by using physical examination data and diagnosis of UC from the Taiwan Cancer Registry Database. Differences in demographic and clinical characteristics among UTUC and non-UTUC groups, UBUC and non-UBUC groups were compared. | 557,063 | Metabolic Syndrome | aged≥40 | both | age, gender, smoking status,  betel quid chewing  behavior,  long-term medication | OR = 1.373, 95% CI= 1.10, 1.70 | | waist circumference >90 cm (35  inch) for men and >80 cm (31 inch) for women; (2) raised  blood pressure (BP): systolic BP ≥130 mmHg or diastolic  BP ≥85 mmHg (3) Raised  fasting plasma glucose (FPG): FPG ≥100 mg/dL (4) higher triglycerides (TG): TG ≥150 mg/dL (5)  HDL-C < 40 mg/dL for men and < 50 mg/dL  for women. |
| Lin, C. C.  2014 | Taiwan | aims to determine cancer risks among patients with type 2 diabetes | A national health insurance program was implemented in March 1995. The datasets of the study consisted of registry for beneficiaries, ambulatory and inpatient care claims, and Registry for Catastrophic Illness from 1996 to 2007 from NHIRD. | 21,680,686 | Diabetes | aged ≥ 20 | both | gender, area registered for NIH program, age, insurance premium | men SIR = 1.07, 95% CI=1.02, 1.13  women SIR = 1.19, 95% CI =1.11, 1.27 | | NA |
| Lo, S. F.  2013 | Taiwan | associated diabetes mellitus (DM) with risk of cancer | The study analyzed the nationwide population-based database from 1996 to 2009 released by the National Health Research Institute in Taiwan | 24,260,000 | Diabetes | 50-75< | both | sex, age, urbanization, hypertension, hyperlipidemia | RR=1.2, 95% CL= 1.13, 1.27 | | NA |
| Lukanova A,  2006 | Sweden | Excess weight has been associated with increased risk of cancer | The Northern Sweden Health and Disease Cohort (NSHDC) is a long-term population-based interventional study intended for the promotion of health in the population of Västerbotten County, northern Sweden. | 74207 | BMI | 29–61 | both | age, calendar year, smoking | Overweight RR= 0.96, 95% CL= 0.48, 1.98  Obese RR =2.12, 95% CL= 0.77, 5.4 | | normal weight (BMI 18.5–24.9), overweight (BMI 25–29.9) and obesity (BMI ≥30) |
| Newton, C. C.2013 | USA | Examined the association between T2DM and bladder cancer incidence | The study in the Cancer Prevention Study II Nutrition Cohort, a large prospective study with information on insulin-use and duration of diabetes. Men and women for this analysis were drawn from  participants in the CPS-II Nutrition Cohort, | 172,791 | Diabetes | 50-75< | both | sex, race, BMI, educational level, alcohol use, smoking status. | RR= 1.01, 95% CI= 0.87, 1.17 | | NA |
| Ogunleye AA, 2009 | Scotland | Diabetes may increase the risk of incidence and mortality from cancer. | In a cohort study using record-linkage health-care datasets for Tayside, Scotland in 1993-2004, we followed up newly diagnosed patients with type 2 diabetes, and two matched non-diabetic comparators, in the national cancer register. | 9577 | Diabetes | 62 | both | deprivation | RR= 0.70, 95%CI= 0.40, 1.21 | | NA |
| Oh SW,  2005 | Korea | assess effects of excess weight on the development of cancers | Our patients were civil servants and private school workers and their dependents, who were members of the Korea National Health Insurance Corporation (KNHIC). this study in a 10-year follow-up cohort of Korean men who were free of prior cancer at baseline | 781,283 | BMI | >20 | men | age, smoking alcohol, regular exercise for more than 30 minutes during a week, family history of cancer, residency area. | Overweight HR =1.14, 95% CI= 0.86, 1.51  Obesity HR= 0.7, men 95% CI= 0.22, 2.19 | | normal (18.50–24.99 kg m−2), overweight (25.00–29.99 kg m−2), obese class I (30.00–34.99 kg m−2), and obese class II and III (⩾35.00 kg m−2) |
| Peila, R.  2020 | UK | examined the association between diabetes, HbA1c, and cancer risk | Briefly, UK Biobank is a large, prospective, population-based cohort study of individuals (54.4% women) who, at the time of recruitment, between 2006 and 2010. | 476,517 | Diabetes | 40–69 | Women | age, non-white race, education, alcohol intake, smoking status, cigarette-years | HR= 1.40, 95% CI=1.10,1.77 | | NA |
| Prizment, A. E.  2013 | USA | Diabetes and risk of bladder cancer | Iowa Women's Health Study | 37,327 | Diabetes | 55-69 | women | age, BMI, WHR, education, smoking status, pack years of smoking, occupation, marital status, physical activity, alcohol use | HR =1.69, 95% CL= 1.19, 2.41 | | NA |
| Rastad, H.  2019 | Iran | assess the association between diabetes mellitus (DM) and the incidence of cancer at different sites | Data from the baseline and first three follow-up visits of the Atherosclerosis Risk in Communities (ARIC) study, an ongoing cohort study of adults from four American communities | 15,118 | Diabetes | 45-64 | both | age, Body Mass Index, Physical activity, alcohol consumption | RR= 0.24, 95% CI= 0.03, 2.16 | | NA |
| Roswall, N.  2014 | European | associations between h, body mass index (BMI), white bladder cancer | European Prospective Investigation into Cancer and Nutrition cohort. Brieﬂy; it includes 23 centers in Denmark,  France, Germany, Greece, Italy, Netherlands, Norway, Spain, Sweden and United Kingdom. Participants  at Basel in (1992–2000), and were mostly recruited from the General population in deﬁned geographical areas. | 390,878 | BMI | 20–97 | both | age, smoking, tumor aggressiveness | Overweight HR=1.04, 95%CI= 0.83, 1.31  obesity HR=1.08, 95%CI= 0.86, 1.36 | | underweight (BMI < 18.5), normal weight (BMI 18.5–  24.9), overweight (BMI 25.0–29.9) and obese  (BMI ≥ 30.0) |
| Rapp,K  2005 | Austria | relation of overweight and obesity with cancer | Incident cancers were identified through the state cancer registry | 145 000 | BMI | 18-94 | both | smoking occupation, increases in relative body weight | Overweight HR=0.9, 95% CI= 0.68, 1.2  Obesity HR=0.94, 95% CI= 0.62, 1.42 | | normal (18.50–24.99 kg m−2), overweight (25.00–29.99 kg m−2), obese class I (30.00–34.99 kg m−2), and obese class II and III (⩾35.00 kg m−2) |
| Rapp, K.  2006 | Austrian | relations between fasting blood glucose and the incidence of cancer | Incident cancer was ascertained by a population-based cancer registry. | 140,000 | Fasting blood glucose | 19-95 | men | smoking, occupational group, BMI | HR=1.11, 95% CL= 0.58, 2.13 | | NA |
| Reeves GK,  2007 | UK | examine the relation between body mass index (kg/m2) and cancer incidence and mortality | In 1996-2001 a total of 1.3 million women aged 50-64 who had been invited for screening for breast cancer at screening centers throughout England and Scotland completed the first study questionnaire. | 1.3 million | BMI | 50-64 | women | age, geographical region, socioeconomic status, reproductive history, smoking status, alcohol intake, physical activity, time since menopause, use of hormone replacement therapy | Overweight RR= 1.15, 95% CL= 0.93, 1.41  Obese RR =1.07, 95% CL= 0.88, 1.3 | | BMI of 25-29.9 as “overweight” and BMI of 30 or more as “obese,”. |
| Russo, A.  2008 | Italy | explore for the first time the link between metabolic syndrome and cancer risk | This study was carried out using Milan’s Health Authority information system, | 16,677 | Metabolic syndrome | 40< | both | NA | males SIR= 1.09, 95% CL= 0.82, 1.43 females SIR= 0.91, 95% CL= 0.44, 1.68 all SIR= 1.06, 95% CL= 0.82, 1.36 | | NA |
| Samanic C,  2006 | Sweden | body mass index to cancer risk | examined the health records of Swedish men who underwent at least one physical examination from 1971 to 1992, and were followed until death or the end of 1999 | 362,552 | BMI | 30-60< | men | age, calendar year, smoking status. | Overweight RR= 0.94, 95% CL = 0.86, 1.03, for Obese RR= 0.91, 95% CL=0.76, 1.09 | | underweight (BMI < 18.5), normal weight (BMI 18.5–  24.9), overweight (BMI 25.0–29.9) and obese  (BMI ≥ 30.0) |
| Stocks, T.  2012 | Sweden | association between blood pressure and cancer risk | Investigated the association in 7 cohorts from Norway, Austria, and Sweden. Participants in Metabolic Syndrome and Cancer Project cohorts took part in health examination(s) between 1972 and 2005 | 577799 | blood pressure | 44 | both | age, body mass index, smoking status | Men HR= 1.12, 95% CL= 1.04, 1.21  Women HR= 0.95, 95% CL= 0.81, 1.11 | | NA |
| Swerdlow, A. J  2005 | UK | cancer risks in a UK cohort patient with insulin-treated diabetes | The Diabetes UK (formerly British Diabetic Association (BDA)) cohort | 28 900 | Diabetes | 30–49 | both | NA | SIR=1.00, 95% CI= 0.61, 1.55 | | NA |
| Teleka, S.  2021 | Sweden | investigated body mass index (BMI) and BP in relation to BC risk | The study included participants from three prospective Swedish cohorts, the Västerbotten Intervention Programme (VIP), the Malmö Preventive Project (MPP) and the Construction Workers Cohort (CWC), | 338,910 | BMI, Hypertension | 38.4 | men | Smoking status. | Overweight RR= .95, 95% CI = 0.79, 1.11  Obese RR=1.11, 95% CI =0.91, 1.36  140-149 mmHg HR=1.02, 95% CI= 0.81, 1.28  150-159 mmHg HR=1.04 95% CI= 0.81, 1.35  ≥160 mmHg HR=1 95% CI= 0.74, 1.33 | | underweight (BMI < 18.5), normal weight (BMI 18.5–  24.9), overweight (BMI 25.0–29.9) and obese  (BMI ≥ 30.0) |
| Tseng, C. H.  2011 | Taiwan | Association between diabetes and incidence of bladder cancer. | Using a large national insurance database. A random sample of 1,000,000 individuals covered by the National Health Insurance was recruited, without bladder cancer at recruitment were followed from 2003 to 2005 | 495,199 men and 503,748 women | Diabetes, hypertension, | >40 | both | age, sex, other potential confounders. | Diabetes RR=1.36, 95% CI, 1.10,1.68 Hypertension RR=0.93 (95% CI= 0.75, 1.16 | | NA |
| Tripathi, A  .2002 | USA | Evaluated prospectively the association of smoking and other potential risk factors with bladder carcinoma incidence in postmenopausal women. | Women participating in the Iowa Women's Health Study completed baseline questionnaires in 1986 and were followed 13 years for bladder carcinoma incidence. | 37,459 | Diabetes, BMI | 55 - 69 | women | age-physical activity, body size, diabetes, alcohol consumption, marital status, and occupation pack-years of cigarette smoking and time since quitting | Diabetes RR= 2.46, 95% CI= 1.32, 4.59  Overweight RR= 0.63 95% CI= 0.33, 1.19  obesity RR= 1.14, 95% CI= 0.86, 1.51 | | NA |
| Walker, J. J.  2013 | Scotland | this study was to use Scottish national data to assess the influence of type 2 diabetes on the risk of cancer at 16 different sites, | All people in Scotland diagnosed with any of the cancers of interest during the period 2001-2007 were identified and classified by the presence/absence of co-morbid type 2 diabetes | men  44,246  women  36,592 | Diabetes | 55-79 | both | age | men RR =1.21, 95% CI= 1.00, 1.47 women RR= 1.42, 95% CI= 1.02, 1.99 | | NA |
| Wolk, A  .2001 | Sweden | Evaluated the relation between obesity and the risks for various forms of cancer. | In a population-based cohort of hospital patients with any discharge diagnosis of obesity during 1965-1993, cancer incidence was ascertained through 1993 by record linkage to the nationwide Swedish Cancer Registry | 28,129 | Obesity | 18-55<x | both | age | SIR = 1.2, 95% CI = 1, 1.6 | | BMI for man  higher than 30.0 kg/m2 and for women BMI higher than  28.6 kg/m2 were as obese; the corresponding  values for overweight are 25.0 kg/m2 and 23.8 kg/m2. |
| Woolcott, C. G.  2011 | USA | Diabetics may have an increased risk for urothelial cancer | Over a median 10.7 years of follow-up, 918 incident cases of urothelial cancer (89% bladder and 11% other urinary tract sites) were identified through tumor registry linkages. | 186,000 | Diabetes, | 45-75 | both | sex, ethnicity, smoking status | all RR = 1.25, 95% CI= 1.04, 1.50). men RR= 1.18, 95% CI= 0.96, 1.47 women RR= 1.48, 95% CI= 1.02, 2.14 | | NA |
| Xu HL, 2015 | china | This study was to investigate the risk of common cancers in Chinese patients with T2DM. | A population-based retrospective cohort study including T2DM patients was conducted in Minhang District of Shanghai, China, during 2004 to 2010. All T2DM patients were enrolled from the standardized management system based on local electronic information system. Newly-diagnosed cancer cases were identified by record-linkage with the Shanghai Cancer Registry. | 36,379 | Diabetes | 21-95 | both | Sex, age. | SIR =1.98, 95% CI= 1.28, 2.68. | | NA |
| Xu, T.  2015 | china | evaluate the impact of body mass on recurrence and progression in patients with Ta, T1 urothelial bladder cancer | Data from patients with Ta, T1 bladder cancer who were treated with transurethral resection of bladder tumor at our center during 2006-2014 were retrospectively studied | 469 | BMI, Diabetes | 67.1 | both | age, smoking status, gender, BMI, hypertension, diabetes mellitus, hypertriglyceridemia, low HDL-cholesterol | Overweight HR= 1.362, 95% CI= 0.571, 3.249 Obesity HR= 3.037, 95% CI= 1.243, 7.420  All HR=3.439, 95% CI= 1.636, 7.229 | | normal weight (BMI < 24 kg/m(2)), overweight (24 kg/m(2) ≤ BMI < 28 kg/m(2)) and obesity (BMI ≥ 28 kg/m(2)) |
| Yood,M,U,  2009 | USA | incidence of melanomas and cancers of the  Colon/rectum, bladder, liver, and pancreas in patients with and without type 2 diabetes. | Using a large national population-based database, conducted a retrospective cohort study of patients with diabetes and a comparison cohort of patients without diabetes. | Diabetes cohort (n = 191,223 patients), non-diabetes comparison cohort (n = 251,489 patients) | Diabetes | 18< | both | age, gender | RR=2.35, 95% CL= 1.76, 3.15 | | NA |

*CI = confidence interval; HR = hazard ratio; OR = Odds ratio; NA = Not available.*
